# Altered Chromogranin A Circulating Levels in Meniere's Disease

**DOI:** 10.1155/2015/643420

**Published:** 2015-04-23

**Authors:** Roberto Teggi, Barbara Colombo, Matteo Trimarchi, Mimma Bianco, Angelo Manfredi, Mario Bussi, Angelo Corti

**Affiliations:** ^1^ENT Division, San Raffaele Scientific Institute, Milan, Italy; ^2^Division of Experimental Oncology, San Raffaele Scientific Institute, Milan, Italy; ^3^Department of Internal Medicine and Division of Regenerative Medicine, Stem Cells & Gene Therapy, San Raffaele Scientific Institute, Università Vita-Salute San Raffaele, Milan, Italy

## Abstract

*Objectives*. Meniere's disease (MD) is an inner ear disorder characterized by episodic vertigo, ear fullness, and hearing loss; usually vertigo attacks cluster in specific period. We studied in MD patients the circulating levels of chromogranin A (CgA) and vasostatin-1 (VS-1), secreted by the neuroendocrine system and involved in the regulation of the endothelial barrier function.* Methods*. Serum levels were assessed in 37 MD patients and 36 controls. The ratio between VS-1 and CgA was calculated.* Results*. CgA was increased in patients compared to controls (1.46 versus 0.67 nM, *p* = 0.01) while no difference was detected for VS-1 (0.41 versus 0.39, resp.). CgA levels in patients positively correlated with the frequency of vertigo spells in the previous four weeks (*p* = 0.008) and negatively with the time in days from the last vertigo attack (*p* = 0.018). Furthermore, the VS-1/CgA ratio negatively correlated with the frequency of vertigo spells (*p* = 0.029) and positively correlated with the time from the last attack (*p* = 0.003).* Conclusion*. The results indicate that variations of CgA levels, but not of VS-1, occur in the blood of patients with active MD, depending on the frequency of vertigo spells and the time from the last crisis.

## 1. Introduction

Meniere's disease (MD) is an inner ear disorder characterized by episodic vertigo associated with low-frequency sensorineural hearing loss, tinnitus, and aural fullness. Commonly accepted pathophysiology includes increased endolymphatic pressure (hydrops). Histopathologic findings in temporal bones support this hypothesis [1], although the relationship between hydrops and MD symptoms remains unproven. Furthermore, according to some authors, hydrops might be an epiphenomenon of MD that arises in end stage as a consequence of a variety of damaging processes of the inner ear [2, 3]. Most MD cases are sporadic and are thought to arise from the interplay of genetic and environmental factors. Normally MD attacks cluster in defined periods, followed by periods of quiescence [4, 5]. Among nongenetic factors, different possibilities might contribute to incite MD relapses, including viruses [6, 7], allergy [8], autoimmunity [9–11], and factors related to fluid and ionic homeostasis [12, 13]. Since MD patients present a higher rate of migraine, some common pathophysiological mechanisms between the two disorders have been postulated [14]. Finally, patients with MD also present a higher rate of psychiatric disorders, above all depression and anxiety [15].

Chromogranin A (CgA) is a 439-residue long protein originally discovered as a major secretory protein of chromaffin cells of the adrenal medulla and was found later to be stored in the secretory granules of many other neuroendocrine cells and neurons [16, 17]. This protein is exocytotically released in circulation, together with the coresident hormone, upon appropriate cell stimulation. Abnormal levels of immunoreactive CgA have been detected in the blood of patients with neuroendocrine tumors or with heart failure, with important diagnostic and prognostic implications [18]. Increased levels have been detected in several other pathological conditions, including renal failure, rheumatoid arthritis, atrophic gastritis, inflammatory bowel disease, and sepsis, or in subjects treated with proton pump inhibitors (a class of drugs commonly used to treat acid peptic disorders) [17–19].

This protein plays an intracellular function in secretory granules biogenesis and hormone condensation and an extracellular function, upon secretion, as a precursor of a variety of bioactive peptides and fragments [16, 17].

CgA may undergo various posttranslational modifications, including glycosylation, sulfation, phosphorylation, and proteolytic processing [17, 20]. A growing body of evidence suggests that CgA fragments exert different and even contrasting biological effects. For example, a peptide corresponding to residues 352–372, called catestatin, acts as a potent inhibitor of nicotinic-cholinergic stimulated catecholamine secretion, induces vasodilation in humans, exerts proangiogenic effects, and plays a cardioregulatory role [16, 21, 22]. A fragment corresponding to the N-terminal region 1–76, named vasostatin-1 (VS-1), inhibits vasoconstriction in isolated blood vessels, is neurotoxic in neuronal/microglial cell cultures, exerts antibacterial effects, regulates cell adhesion, depresses myocardial contractility/relaxation, counteracts the *β*-adrenergic-induced positive inotropism, and modulates coronary tone [17, 23]. In addition, VS-1 is a potent inhibitor of endothelial cell activation caused by cytokines, enhances the endothelial barrier function, protects vessels from TNF-induced vascular leakage, and exerts antiangiogenic effects in various in vitro and in vivo assays [23–26]. CgA has been demonstrated to be expressed in different portions of inner ear of guinea pig, including organ of Corti, utricle, and saccule [27]. Moreover, other investigators showed the presence of catestatin in the auditory and vestibular pathways, suggesting its possible role as a neuromodulatory peptide [28].

Based on the notions that CgA and VS-1 can play important roles in the endothelial barrier function and vascular homeostasis and that altered fluid homeostasis might represent a pathogenetic event in MD, we investigated the circulating levels of these polypeptides in MD patients. We found that the relative levels of VS-1 and CgA change in MD depending on the frequency of vertigo spells and the time from the last attack.

## 2. Materials and Methods

### 2.1. Study Population

The study population included 37 consecutive MD patients (24 females, mean age 45.3 ± 10.8 years), recruited at the Outpatients Clinic of the Vestibular Disorders Unit at San Raffaele Hospital, Milan, Italy. All patients fulfilled all AAO-NHSF criteria for definite MD according to AAO-HNS guidelines [29] and were selected when referring at least with one attack of vertigo in the last 30 days. The study was approved by the Ethics Committee of the San Raffaele Scientific Institute and all subjects signed informed consent. Exclusion criteria were clinical history of therapies with proton pump inhibitors or serotonin selective reuptake inhibitors in the month before recruitment and a clinical history of heart, hepatic, or renal failure.

The following clinical data were recorded for each MD patient: (a) history of hypertension or migraine and first attack of migrainous headache; (b) familial history of episodic vertigo of any kind; (c) positivity for one of the following autoantibodies: nucleus, smooth muscle, mitochondria, thyroid, cardiolipin, beta 2 glycoprotein, lupus-like anticoagulant, and rheumatoid factor; (d) time from the last attack of vertigo (in days) and number of attacks in the last 30 days; and (e) age at the first vertigo attack.

Control subjects (*n* = 36) referred with a negative clinical history for vertigo and no clinical conditions were known to be associated with increased levels of CgA. They were selected with gender distribution and mean age overlapped with patients (mean age 43.9 ± 12.6 years; 22 were females). Exclusion criteria included treatment with proton pump inhibitors or serotonin selective reuptake inhibitors in the 30 days before blood collection.

In subjects of both groups a body mass index has been calculated; sex, age, and body mass index (BMI) of control subjects and MD patients are reported in Table 1.

### 2.2. CgA- and VS-1-ELISAs

Blood samples from control subjects and patients were collected into tubes without anticoagulants (Becton Dickinson, France), immersed in ice, and immediately transported to the laboratory for processing. Blood was allowed to clot for 30 min, centrifuged (3000 ×g, 10 min), and stored at −20°C.

*CgA-ELISA*. Serum levels of CgA were detected using a sandwich ELISA, previously described, based on the use, in the capture step, of monoclonal antibody (mAb) B4E11 against an epitope located within CgA residues 68–71 and, in the detection step, of a rabbit anti-recombinant CgA polyclonal antibody against epitopes located in central region of CgA (immunodominant epitopes 90–133, 163–187, 222–256, and 315–338). This assay recognizes full-length CgA1-439 and fragments containing the N-terminal and part or all central regions (e.g., CgA1-436, CgA1-409, CgA1-400, and CgA1-373), but not CgA1-76 (VS-1) [25]. This ELISA has been previously used for CgA detection in the blood of patients with cancer, heart failure, renal failure, rheumatoid arthritis, or other diseases [19, 26]. 

**Figure 1:**
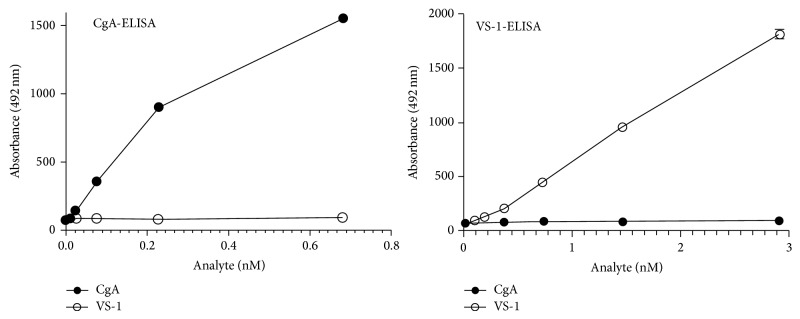
Dose-response curve of CgA-ELISA and VS-1-ELISA to recombinant human CgA and VS-1.

*VS-1-ELISA*. Serum levels of VS-1 were detected using a sandwich ELISA, previously described, based on the use of mAb 5A8 (against an epitope located within residues 53–57 of CgA) in the capture step and a rabbit polyclonal antibody raised against the peptide KERAHQQ (with a free carboxyl group) coupled to Keyhole Limpet Hemocyanin in the detection step [25]. This antibody, which is against a peptide that corresponds to the C-terminal sequence of CgA1-76 (VS-1), can recognize VS-1, but not the full-length CgA precursors. Consequently, the VS-1-ELISA can detect VS-1, but not larger fragments or full-length CgA. The use of this combination of antibodies in a sandwich ELISA has been previously reported for the detection for VS-1 in the sera of patients with sepsis [26]. The dose-response curves of CgA-ELISA and VS-1-ELISA showing the assay specificity and dynamic ranges for these analytes are shown in Figure 1.

### 2.3. Statistical Analyses

Continuous variables are described as mean and standard deviation. The significance of difference between groups was evaluated by *t*-test for independent samples. A Mann-Whitney test was performed when data did not present a normal distribution. A Spearman test was performed to investigate the association between CgA, VS-1, and ratio values and migraine, hypertension, familial history of vertigo, positivity for autoantibodies, frequency of vertigo spells in the last month, and time (in days) from the last attack of vertigo; finally, a general linear model was performed to assess the independent role of age, BMI, and frequency of vertigo spells in CgA levels.

## 3. Results

Five out of 37 MD patients presented arterial hypertension (14%), 16 had a diagnosis of migraine (43%), 11 were positive for at least one of the autoantibodies tested (30%), and 9 referred with a familial history of episodic vertigo (24%). The age of the first attack of vertigo was 41.2 ± 9.8 years. The mean value of time from the last attack of vertigo was 15.6 ± 8.6 days; the mean value of number of spells in the last 30 days from blood collection was 3 ± 1.9.

**Figure 2:**
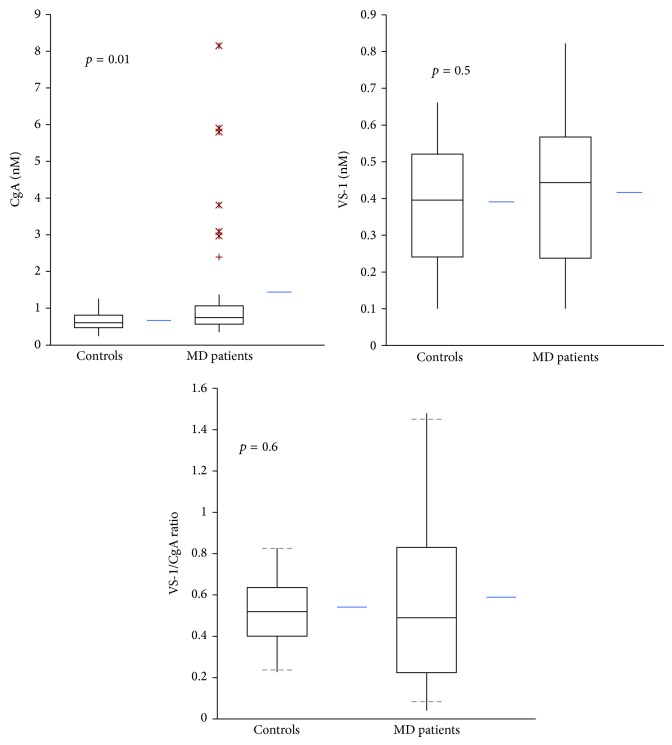
Serum levels of CgA, VS-1, and VS-1/CgA ratio in normal subjects (*n* = 37) and MD patients (*n* = 37).

The circulating levels of CgA, VS-1, and the VS-1/CgA ratio in patients and controls are reported in Table 2 and Figure 2. MD patients presented higher serum levels of CgA, while no significant difference was observed for VS-1. Notably, some patients had abnormally elevated levels of circulating CgA.

**Figure 3:**
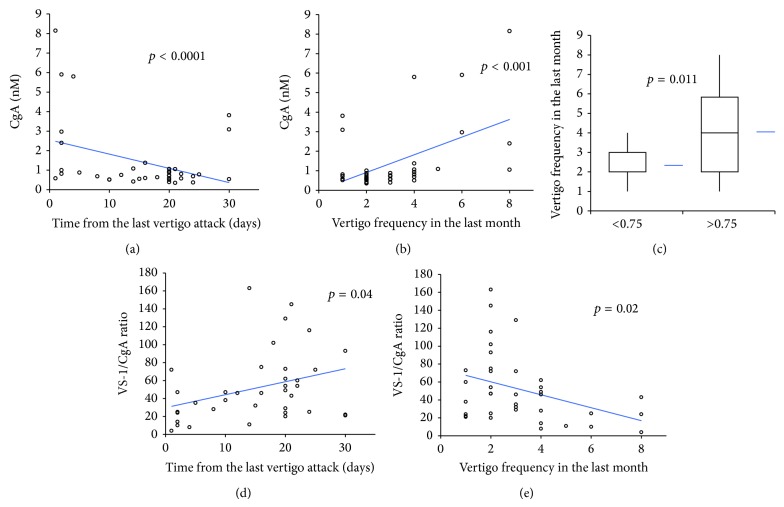
Correlation of CgA or VS-1/CgA ratio with time from last vertigo attack or number of attacks in the last month (a, b, d, and e). Number of vertigo attacks in MD patients with CgA < 0.75 nM or >0.75 (median value) (c).

Since CgA values in patients did not show a normal distribution, a Spearman test was performed to assess the correlation of CgA with other clinical variables. Serum levels of CgA positively correlated with the vertigo frequency occurring within 30 days before blood collection (rs stat 0.43; *p* = 0.008) and negatively correlated with the time in days from the last vertigo attack (rs stat −0.39; *p* = 0.018) (Figure 3). Notably, subjects with values greater than the median CgA value (0.75 nM) had a higher number of vertigo spells in the last month (4 ± 2.4 versus 2.3 ± 0.8, *p* = 0.011) (Figure 3). No correlation was observed between CgA levels and migraine, hypertension, positivity of at least one of autoantibodies, and familial cases of episodic vertigo. Moreover a general linear model test demonstrated no association between CgA levels and BMI (*p* = 0.44) or age (*p* = 0.55). Moreover, every increase of a single attack determined an increase of CgA value of 0.363 (*b* = 0.363; SD 0.101).

No correlation was observed between VS-1 serum levels and number of crises in the last month and time from the last attack. Consequently we observed a negative correlation between the VS-1/CgA ratio and vertigo frequency in the last 30 days (Spearman; rs = −0.36; *p* = 0.029) and a positive correlation with the time in days from the last crisis (rs = 0.47; *p* = 0.003) (Figure 3). No correlation was observed between these clinical variables and clinical history of migraine, hypertension, familial cases, and positivity for at least one autoantibody.

## 4. Discussion

The results show that variations of CgA levels, but not of VS-1, occur in the blood of patients with active MD, depending on the frequency of vertigo spells and the time from the last crisis. In particular, the results show that the serum levels of CgA, but not of VS-1, correlate with the number of crises in the last 30 days from blood collection and the time from the last vertigo attack, being higher in patients with a higher number of vertigo spells in the last month and in patients referring with more recent attacks. Considering that CgA is stored and coreleased with serotonin and other catecholamines by cells of the neuroendocrine system [30], the increased circulating levels of CgA observed in MD patients referring with a high number of vertigo spells in the last month may be related to activation of the autonomic system (in which serotonin has been demonstrated to be an important neurotransmitter) consequent to vestibular overloading [31]. Furthermore, since serotonin is present in the inner ear and vestibular nuclei of the rat [32], we cannot exclude that repetitive vestibular stimulation and the possible consequent release of CgA in these brain areas might contribute to enhancing the circulating levels of this protein. At variance no difference has been detected in the VS-1 levels of controls and patients. As a consequence, a marked reduction of VS-1/CgA ratio occurs in MD patients with higher recurrence of vertigo spells. Furthermore the VS-1/CgA ratio positively correlates with the time from the last attack, pointing to a possible change in the proteolytic processing of CgA or to differential secretion or metabolism of these polypeptides in patients with increased clustering of vertigo. Given that VS-1 and CgA fragments can exert complex and even opposite effects in the homeostatic regulation of the endothelial lining of vessels [26], these findings may have important pathophysiological implications. For example, it is possible that CgA/VS-1-dependent changes in the homeostatic mechanisms related to endothelial barrier may have a role in hydrops and, consequently, in vertigo clustering attack.

Further studies are necessary to verify this hypothesis. Although this is a descriptive study performed on a small sample size and without serial samples (which represent important study limitations), our results may stimulate new studies on a larger cohort of MD patients aimed at assessing whether the detection of circulating CgA has a predictive value regarding vertigo recurrence.

## Figures and Tables

**Table 1:** Demographic data (sex and age) and body mass index (BMI) of normal subjects and MD patients.

Subjects	(*n*)	Sex	Age	BMI
Controls	*36 *	22 females	43.9 ± 12.6	24.98 ± 3.63
MD patients	*37 *	24 females	45.3 ± 10.8	24.26 ± 2.76
(p value)		*ns *	*ns *	*ns *

**Table 2:** Circulating levels of immunoreactive CgA and VS-1 in normal subjects and MD patients.

		ELISA assay		
Subjects	(*n*)	CgA	VS-1	VS-1/CgA
		(nM)	(nM)	(%)
Controls	*36 *	0.67 ± 0.27	0.39 ± 0.18	54 ± 19
MD patients	*37 *	1.46 ± 1.79	0.41 ± 0.19	52 ± 38
(*p* value)		*(0.01) *	*(0.5) *	*(0.6) *
